# In-Tire Distributed Optical Fiber (DOF) Sensor for the Load Assessment of Light Vehicles in Static Conditions

**DOI:** 10.3390/s21206874

**Published:** 2021-10-16

**Authors:** Martin Fontaine, Alex Coiret, Julien Cesbron, Vincent Baltazart, David Bétaille

**Affiliations:** 1 COSYS-SII, Université Gustave Eiffel, IFSTTAR, 44344 Bouguenais, France; alex.coiret@univ-eiffel.fr (A.C.); vincent.baltazart@univ-eiffel.fr (V.B.); david.betaille@univ-eiffel.fr (D.B.); 2 CAPTELS Society, 34270 Saint-Mathieu-de-Tréviers, France; 3 UMRAE, Université Gustave Eiffel, IFSTTAR, CEREMA, 44344 Bouguenais, France; julien.cesbron@univ-eiffel.fr

**Keywords:** wheel load, intelligent tire, fiber optical, load evaluation, tire contact patch length

## Abstract

Modern vehicles are using control and safety driving algorithms fed by various evaluations such as wheel speeds or road environmental conditions. Wheel load evaluation could be useful for such algorithms, particularly for extreme vehicle loading or uneven loads. For now, smart tires are only equipped by tire pressure monitoring systems (TPMS) and temperature sensors. Manufacturers are still working on in-tire sensors, such as load sensors, to create the next generation of smart tires. The present work aims at demonstrating that a static tire instrumented with an internal optical fiber allows the wheel load estimation for every wheel angular position. Experiments have been carried out with a static tire loaded with a hydraulic press and instrumented with both an internal optical fiber and an embedded laser. Load estimation is performed both from tire deflection and contact patch length evaluations. For several applied loads from 2800 to 4800 N, optical fiber load estimation is realized with a relative error of 1% to 3%, almost as precisely as that with the embedded laser, but with the advantage of the load estimation regardless of the wheel angular position. In perspective, the developed methodology based on an in-tire optical fiber could be used for continuous wheel load estimation for moving vehicles, benefiting control and on-board safety systems.

## 1. Introduction

### Context and General Aim

Vehicle load measurement is a key technological issue, especially in automotive and road freight fields. The health of roads and infrastructures, fuel consumption and driving safety are all linked to the vehicle load. Actually, infrastructure sustainability is highly related to wheel loading, especially for extreme temperature situations, since rutting or frost issues are amplified by high loads. Fuel consumption, as well as tire wear and wearing of automotive parts, are also load dependent. Finally, driving safety, according to all previous assertions, is mainly based on vehicle control. From a load point of view, a specified weight distribution on axles is mandatory to ensure a good equilibrium of forces at the tire-to-road contact patches. Until now, the driver’s ability is required to evaluate his/her vehicle load distribution.

Reflections on new transport solutions, such as autonomous vehicles or truck platooning and energy savings, enforce us to think about load monitoring, especially for autonomous vehicles, without a driver. To measure this load, the vehicle tires appear to be suitable because of their high deformation behavior under loading, and the avoidance of intermediate behavior in the case of the instrumentation of axles or dampers. For now, in-tire load sensors in the state-of-the-art are not designed to sense load in a non-rolling condition for any wheel angular position.

The present work aims at introducing a new embedded load sensor for a non-rolling vehicle whatever the wheel angular position. Indeed, it is of importance for an autonomous or driven vehicle that the load and its distribution could be checked before taking the risk of starting travel with inadequate load conditions. The proposed sensor is based on an optical fiber bonded along the tire inner liner circumference. This new sensor makes it possible that the spatial circumferential strain profile measurement for a non-rolling vehicle. From this profile, the vertical load applied onto the tire is computed.

This paper is organized as follows: a state-of-the-art on main measurable quantities for the load measurement, namely deflection first, and second the circumferential strain and contact patch length (CPL), is exposed. Models and equations to compute the load from these quantities are also exposed in that section. This showcases the novelty of the proposed sensor, especially for the static vehicle load sensing. Then, in the next section, the Distributed Optical Fiber Sensor (DOFS) specification and the instrumented test bed are introduced. Finally, the load estimation method is exposed and the results are discussed.

## 2. Literature Review/State-of-the-Art

### 2.1. Load Sensing: From Commercial Use to Autonomous Vehicles

Historically, load sensing has mainly been used in transportation for enforcement purposes, to control axles’ weight limits applied on roads, or for billing purposes, to establish the value of raw materials loaded on a given heavy carrier.

Large weighting platforms, or scales, are still widely used by manufacturers in those aims [1]. These platforms can either be embedded in roads or be portable. Studies have also been worked out to extend investigations on 3D forces, for discrete matrix forces arrays in low speed cases [2] or aggregate forces in high speed cases [3]. Vehicle manufacturers and vehicle fleet managers have developed embedded load sensing systems. Such systems involve sensors located on arms, dampers [4] or on suspension blades [5,6], on axles [7] or even, indirectly, at the truck to trailer hitch [8]. Even if new vehicle-based systems have appeared, the main aim was still related to weight enforcement and goods billing.

Nowadays, the emergence of autonomous vehicles is bringing a new need related to vehicle loading: without driver, the knowledge of an autonomous vehicle load is of great importance to adapt braking strategies and to enhance stability models in curves. Further than the total load, the knowledge of each wheel loading can provide information on loading distribution and alert an operator about stability issues (or limit the allowed speed).

Simultaneously, research on in-tire sensors has been expanding for years. Manufacturer’s concepts are also emerging and suggest that intelligent tires are going to be a reality in few years. Small sensors can be used inside tires without impacting the tire behavior and safety too much, and low-power, wireless sensors are usable without impact tire sealing. Micro Electro Mechanical Systems (MEMS) have been used in [9] in order to measure accelerations at a point inside a tire and to estimate load, pressure and even friction coefficients. Nevertheless, estimation models as a brush model are subjected to fine tuning.

A large literature review of the MEMS use in transportation is part of research on heavy vehicle safety [10]. This experimental work is focused on critical accelerations monitored by MEMS on road cargo. The preliminary literature review shows that MEMS can be used for the measurement of actual roadways’ states, or to estimate mutual influence of roads and vehicles, or to be used to feed automatic warnings in the case of an accident, or for smart tire measurements, or for the indication of cargo dommage [10]. The common advantage of MEMS for all these application is to monitor forces with a small device at any location. In fact, vehicle dynamic and static contact features, such as vertical load, can be described by the tire behavior, but measurement and estimations could be more precise by using larger measurement scales, as was chosen in this present work. Among measurable quantities that described this behavior, circumferential strain, deflection and contact patch size are mainly studied to evaluate these features and more specifically the load. The following subsections provide an overview of the state-of-the-art on these measurable quantities and of the background to understand the load sensing method proposed in this work, which involves an instrumented tire in order to address the loading knowledge issue from the vehicle.

### 2.2. Deflection Measurement and Load Relationship

The tire deflection is the vertical displacement of the wheel axis due to the wheel loading [11]. The deflection value is obtained by sensing the distance between the wheel axis and the ground. It is also called the static loaded radius and can be measured by in-tire sensors from the rim to the inner liner. Tire deflection is mainly used by standards organizations, such as ETRTO (European Tyre and Rim Technical Organization), to control and set the load capacity of each tire.

In the literature, several studies aim to sense the deflection from a wheel-embedded sensor. Some examples are presented in Table 1.

**Figure 1:**
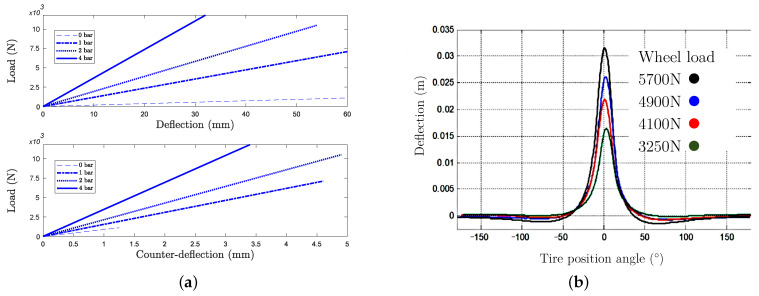
(**a**) Deflection and counter deflection of a tire for 0–12,000 N loadings. (**b**) Tire deflection measurement over tire circumference, adapted from [16].

The most common sensors used are the ultrasonic sensors [12,13] and the laser sensors [14,17]. Besides the well known tire main deflection, occurring at the bottom part of the tire, there exists a little known counter deflection which occurs at the upper part of the tire, as a repercussion of the main deflection and at a much lower amplitude of typically one to ten. As an illustration, tire deflections and counter deflections are plotted in Figure 1a from experimental data [18], which have been simplified by linearization.

In [16], a position sensitive diode (PSD) is used with an infrared diode glued on the inner side of the tire to measure the deflection over the tire circumference and for various loads (Figure 1b). This profile has been obtained by rotating the tire during the sampling for a set of vertical load values. The maximum displacement value at the center corresponds to the tire main deflection.

**Figure 2:**
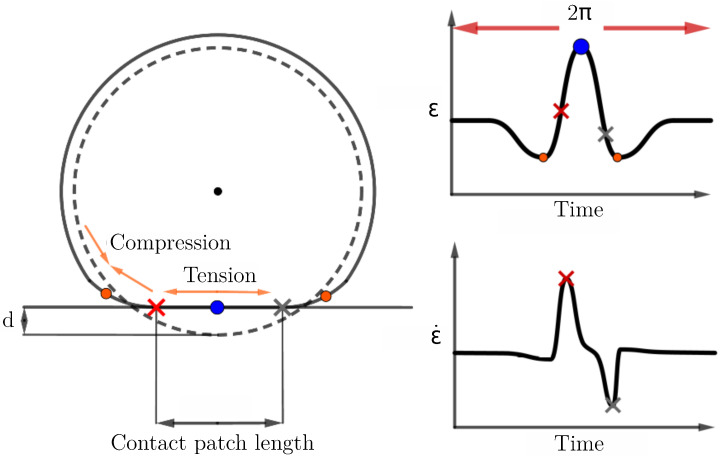
Schematization: tire tension and compression zones (**left**) and typical dynamic circumferential strain measurement (**right**, **up**) and strain rate (**right**, **down**), adapted from [19].

According to Figure 1, static flattening induced by loading is subjected to a maximum deflection value that varies according to internal pressure conditions. It also highlights that a counter deflection exists at the topside of the tire and is around one tenth of the tire deflection. The deformation principle involved by the generation of this counter deflection is presented in [19,20,21] and it is drawn here in Figure 2. In this figure it can be seen that the deformation of the curved wheel base into a flat surface results in a tension over the contact patch (blue dot), surrounded by compression areas (red dots); these compressions of the tire belt secondarily result in a counter deflection of the tire topside, by belt wire traction.

A linear load-deflection relationship is clearly identified in the literature. From a mechanical point of view, it is currently assimilated to a spring model [22], with a vertical stiffness $K_{z}$. The vertical load is thus expressed as follows:

(1) $$F_{z}=K_{z}*d,$$

with $F_{z}$ (in N) the vertical load and d (in mm) the deflection of the tire. The stiffness value $K_{z}$ is expressed in N/mm or kg/mm and is affected by the vertical structural stiffness of the tire sidewalls and the inflation pressure.

Several ways can lead to the vertical stiffness estimation such as flexible ring models, semi-empirical relationships or load capacity formulas from standards. The Formula (2) of the vertical stiffness, expressed in N/mm, was obtained empirically from load/deflection measurements over a wide range of tire sizes in [18].

(2) $$K_{z}=0.00028Pg*\sqrt{W*OD}+K_{zsw},$$

with the internal pressure *P* (in kPa), the tire footprint *W* (in mm) width, outside diameter (*OD*, in mm) and g= 9.81 m/s^−2^ the acceleration of gravity. The $K_{zsw}$ constant represents the structural stiffness, that is, the tire stiffness value for a deflated tire. The $K_{zsw}$ equals 33.84 N/mm (3.45 kg/mm) in [18]. In [23], Equation (2) is modified to express the tire footprint width only with tire geometric values as follows:

(3) $$K_{z}=0.00028Pg*\sqrt{\left(-0.004a_{r}+1.03\right)S_{N}*OD}+K_{zsw},$$

with $a_{r}$ as the aspect ratio and $S_{N}$ the nominal section width of the tire. In this study, Equation (3) is used to compute the load from the measured deflection in Section 5.

### 2.3. Strain Measurement and Load Relationship

Material strain is defined as the elongation ratio along a direction. Some of the main studies on the tire strain measurement are listed in Table 2. It provides an overview on methods and sensors used. Strain-load relationships or observations in each article are also exposed.

According to this table, sensing the tire strain from the inner liner in the circumferential direction provides a more sensitive strain value than the other directions [21,26,28]. In-tire strain transducers are usually dependent of the angular position of the tire. In fact, to establish the tire strain along the circumference, tire rotation is mandatory. This leads to dynamic perturbations such as braking, acceleration or dynamic forces, which cause fluctuations in the strain value.

A typical circumferential strain profile is schematized in Figure 2.

The strain time signature can be split into three distinct parts. In the central part, the elongation caused by a tension of the inner liner is observed as explained in [28]. Compression is then observed at both sides of the central part. The third part corresponds to the upper part of the tire which is not influenced by the load. In theory, this profile is symmetric about the center of the contact patch length where the maximum strain value is observed. However, in practice, driving conditions, braking, acceleration or dynamic forces can generate an asymmetric profile [19]. As shown in Table 2, the time derivative of this strain profile, the so-called strain rate, is mostly used to assess the contact patch length (CPL). The length corresponds to the distance between the peaks of the strain rate according to Figure 2. This method is based on the Morinaga assumption [29] which defines the CPL measured from the inner liner as the distance between the points where the force variation in the radial direction is the greatest, which corresponds to peaks in the strain rate profile. According to Table 2 and Table 3, the CPL knowledge is suitable to compute the vertical load through models.

### 2.4. Acceleration Measurement and Load Relationship

Inner liner acceleration sensing during rolling is also a suitable way for estimation of the contact patch length and associated forces. Studies on this aspect are listed in Table 3. Measurement of acceleration can be performed in several directions (radial, circumferential or transversal) and realized by accelerometers placed on the inner liner of the tire. As for strain sensing, the contact patch length is estimated from the acceleration profile. According to Table 3, the method for contact patch length computing (i.e., peak to peak or zero-crossing) depends of the sensor direction (i.e., radial or circumferential).

Radial displacement can also be estimated and used in a flexible ring model [30] or recursive least square fitting [31] to compute the vertical load.

## 3. Experimental Setup

Whether with laser, accelerometers, strain gauges or ultrasonic sensors, for load evaluation means investigated up to now in the literature, the static measurement requires turning the considered wheel at a given angular position. An immediate measurement on a vehicle, before each journey, is therefore not easy, especially if the measurement involves several tires. To solve this, a distributed strain sensor made by an optical fiber along the tire inner liner circumference is proposed in this work, with the advantage of delivering the strain all along the belt at each measurement step. Preliminary results can also be found in [35].

This work, as a proof of concept, is focused on a static tire. The DOF sensor gives the opportunity to evaluate the tire load independently to the tire rotation angle. Therefore the experimental setup is built around a non rotating tire. However, there exist extension perspectives of this work on the rotating tire while surpassing current limitations. Firstly, the fiber bonding has to be studied more precisely in order to stand up to the tire dynamic forces which could cause damages on the bonding and the fiber sensor. Secondly, the sensor interrogator should be miniaturized to be embedded in the wheel or a rotary coupler should be used [24]. In an intermediate way, we can imagine a smart tire, equipped with a circumferential optical fiber sensor, which can be plugged to an external interrogator, at the start of a journey; for example, in order to provide the vehicle load.

### 3.1. Vertical Loading Device

**Figure 3:**
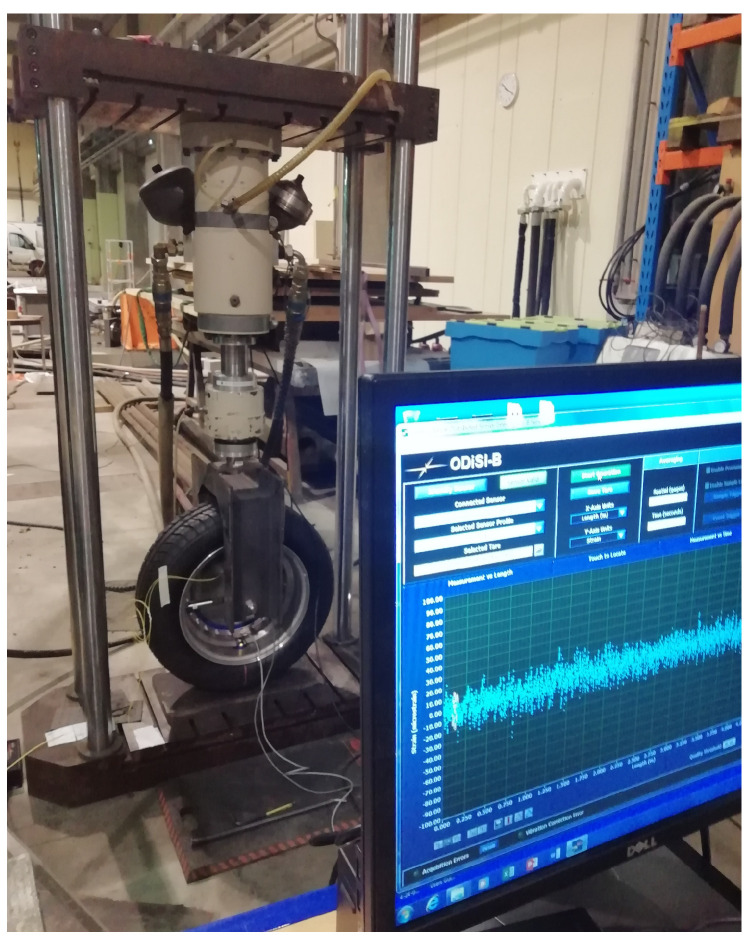
Experimental setup with the instrumented tire and the hydraulic press.

**Figure 4:**
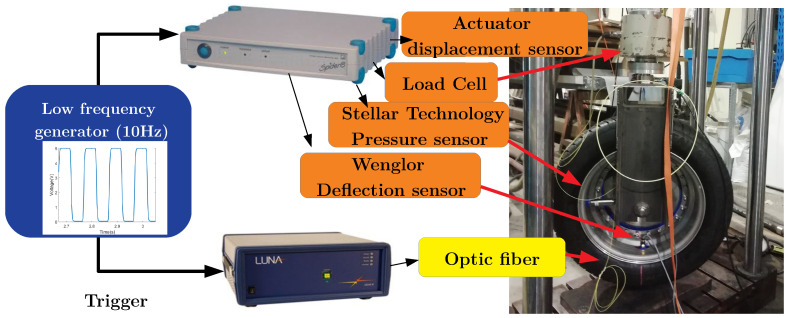
Synchronized data sampling for optical fiber (Luna system) and other sensors (Spider8 Digital Analog Converter).

The experimental setup consists of a test wheel submitted to a vertical load applied by an hydro-static piston, both being constrained in displacement by a loading cage composed of top and bottom plates interlocked by a four columns cage (force press; Figure 3 and Figure 4).

Vertical forces are applied by the hydro-static piston and are controlled via a loading cell (sensitivity of 0.2 mV/kN, displacement range of 150 mm).

### 3.2. Tested Wheel

The wheel is instrumented with the following sensors:

- a laser beam for the measurement of the deflection (Wenglor, sensitivity 0.2 V/mm). The laser head is screwed on the tire rim and points radially to a white target painted on the center of the inner side on the tire contact patch;
- a pressure sensor for the measurement of the tire inflation pressure (Stellar Technology, sensitivity 2.9 V/bar);
- an optical fiber glued in the middle of the inner tire belt over the full tire circumference (Luna system).

The tire used during the tests was new. Its dimensions, loading index and speed rating are 185/65R15 88T. It is manufactured by GoodYear, with a radial type construction, 1 polyester ply in the sidewall, and 1 polyester/2 steel/1 polyamide plies in the tire belt. The tire inflation pressure is set at 2.1 bar.

Two acquisition systems are simultaneously used to record the data. The general conditioning and analog to digital converter system (Spider8) is used to acquire the data of all the sensors except the optical fiber sensor, at a common sampling frequency of 400 Hz.

The optical fiber has its own acquisition system, manufactured by Luna, the ODISI-B interrogator. According to the manufacturer, the chosen optical fiber and the ODISI-B interrogator allow a maximum strain measurement of about 10,000 μϵ (1%). Our experiments, with a large loading scale, lead to a maximum of 4000 μϵ, so the tire maximum strain is within the DOF sensor strain scale. The DOF sensor acquisition is made at a sample frequency of 10 Hz.

A low frequency generator allows the synchronization of the two acquisition systems. A general view of the experimental system is provided in Figure 4.

### 3.3. In-Tire Dof Sensor

DOF sensors allow the continuous determination of deformations all along a single optical fiber. These sensors are based on reflectometry such as Rayleigh scattering by involving swept-wavelength interferometry [36]. They are widely used for Structural Health Monitoring (SHM) for deformations checking all along a monitored element (e.g., [37]).

The fiber index of refraction is locally altered by the surrounding strain and temperature variations. Fluctuation of this index induces signal losses by back scattering, namely, the Rayleigh scattering phenomenon. Practically the local strain or temperature variations are determined by comparing the stressful scatter frequency profile to the stressless scatter frequency profile, or the reference profile. The spectrum frequency shift can be expressed as a function of the temperature and strain variations:

(4) $$\frac{\Delta \nu }{\nu }=K_{T}\Delta T+K_{\epsilon }\Delta \epsilon ,\;$$

with $\frac{\Delta \nu }{\nu }$ as the frequency shift, ΔT and Δϵ respectively the temperature and strain variations, and $K_{T}$ and $K_{\epsilon }$ constants of proportionality.

**Figure 5:**
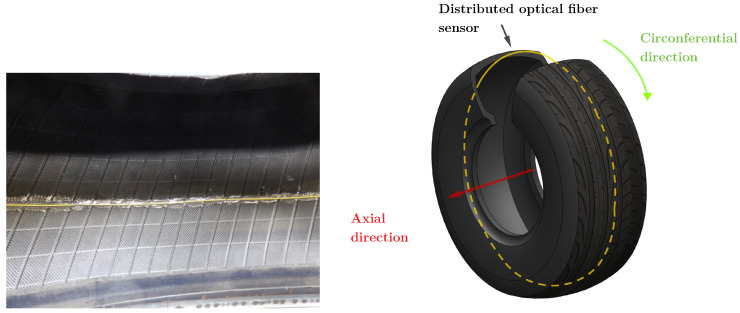
Optical fiber (in yellow) bonding on the tire inner liner (**left**) and schematic view of the optical fiber implementation in the tire (**right**).

If the tests are performed at a constant temperature, the strain can be directly computed. The DOFS method allows to locally evaluate the strain at each spatial pitch of the optical fiber, down to pitches of 0.65 mm. Up to 1500 strain values can be recorded all along a 1 meter long fiber. The optical fiber is bonded along the inner liner perimeter of the tire as shown in Figure 5. The optical fiber is going through the rim by a cable gland to ensure the pressure sealing. The chosen paste is a bi-component epoxy paste (Araldite 2011) well adapted for rubber bonding. Agreement between measured strain values (Section 5) and state-of-the-art results confirms this choice.

Moreover, before each loading experiment, the operator performed a calibration of the fiber response beforehand to cancel any potential offset. Sometimes successive loading may have alter the fiber response at some locations, because of rare loss of adhesion of fiber to tire, either due to fiber–epoxy or epoxy–tire contact. These points are easily detected on the circumferential fiber signatures and filtered out of the around 3000 other measurement points.

## 4. Data Processing for Load Assessment

Through this section the data processing methodology for load assessment is exposed. Firstly, the strain profile measured from the DOF sensor is presented. Then the processing of data is presented and applied on a loading case in order to calibrate the prediction formula. In the same way, the deflection measurement is exposed and the data processing to compute the load is applied.

### 4.1. Loading Cases

**Figure 6:**
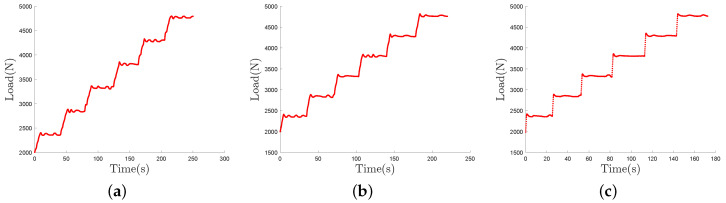
Step loading cases considered in the study: slow (**a**), intermediate (**b**) and fast (**c**) loading speed, respectively denoted $L_{50}$, $L_{100}$ and $L_{500}$.

As described in Section 3, the instrumented wheel has been loaded by means of an hydraulic press. Three loading cases have been considered for the tests as shown in Figure 6. Each test consists in a 6-steps loading between 2000 N and 4750 N. The step duration is the same for each test and roughly equal to 30 s. The difference between the three tests is the loading speed between each step. Thus, Figure 6a–c respectively correspond to a slow, intermediate and fast loading speed. Loading speed is respectively equal to 50 N/s, 100 N/s and 500 N/s for the slow, intermediate and fast loading speed tests. In the following these loading cases will be referred to as $L_{50}$, $L_{100}$ and $L_{500}$.

**Figure 7:**
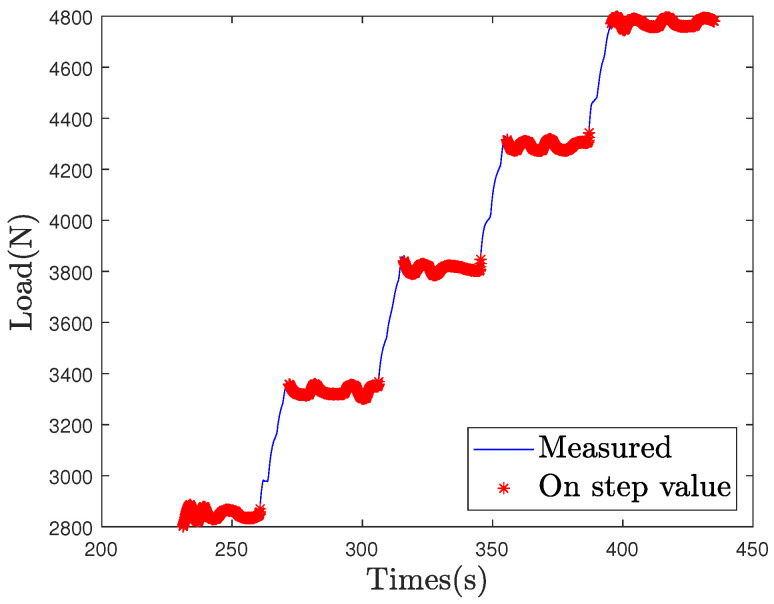
Identified load step values on the $L_{50}$ loading case.

The static load estimation from the DOF sensor and the laser sensor is based on load formulas, exposed in Section 4.2 and Section 4.3. The $L_{50}$ loading case is used as a calibration test in order to estimate formula constants. Only measured values on loading steps are selected to proceed to the calibration. The loading steps are defined using the ‘findchangepts’ Matlab function. The selected loading steps values are exposed in the Figure 7.

### 4.2. Load Computation from Deflection Data

For the sake of comparison, the load computation from a deflection measurement is processed.

Once the deflection is sensed, a linear relationship is used to compute the load. As exposed in the Section 2.2, establishing load-deflection relationship requires vertical stiffness knowledge. In this article, the stiffness formula defined in [18] and precised in [23] is applied to compute the vertical load.

It relies on Equation (1) which is written as follows:

(5) $$F_{z}=\left(K_{zP}+K_{zsw}\right)\left(d_{laser}-d_{0}\right),$$

with $K_{zP}=0.00028Pg\sqrt{\left(-0.004a_{r}+1.03\right)S_{N}*OD}$ the tangent stiffness part is expressed in N/mm, due to tire inflation and g= 9.81 m/s^−2^ the acceleration of gravity. For the tested tire, the aspect ratio $a_{N}=0.65$ and nominal section width $S_{N}=185$ mm. Term $K_{zsw}$ is the tangent stiffness part (in N/mm) due to the sidewalls of the tire, while $d_{laser}$ is the displacement measured by the laser sensor (in mm) and $d_{0}$ is a correction factor (in mm) due to possible alignment error of the laser sensor. Thus, the deflection *d* is given by:

(6) $$d=d_{laser}-d_{0}.$$

**Figure 8:**
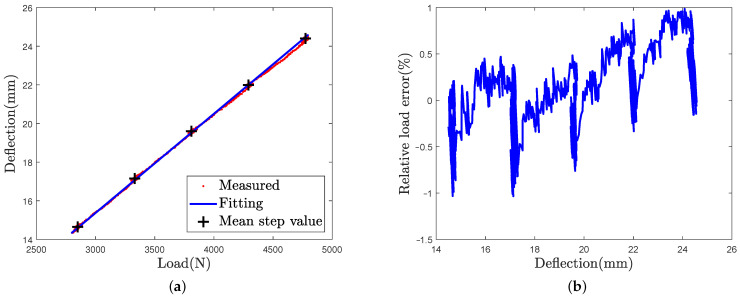
(**a**) Tire deflection measured in red and its fitting curve in blue, (**b**) Relative error on the predicted load.

As the $K_{zsw}$ constant value is defined in [18] for a large range of tires, its value is evaluated for the tested tire in order to better fit to the real structural stiffness of the sidewalls. In order to estimate parameters $K_{zsw}$ and $d_{0}$ for the tested tire, the $L_{50}$ loading case deflection mean value on step are fitted with a linear fitting curve. The deflection and its fitted curve as a function of the vertical load are shown in Figure 8a. The error between the measured deflection and the fitted curve is represented in Figure 8b. The absolute fitting error is less than 1% and the highest values are mainly observed during load-step transitions. The coefficients estimated from the fitting are $d_{0}=1.27$ mm et $K_{zsw}=18.01$ N/mm (=1.83 kg/mm). These coefficients are used in the following in Section 5.

### 4.3. Load Computation from Dof Strain Data

**Figure 9:**
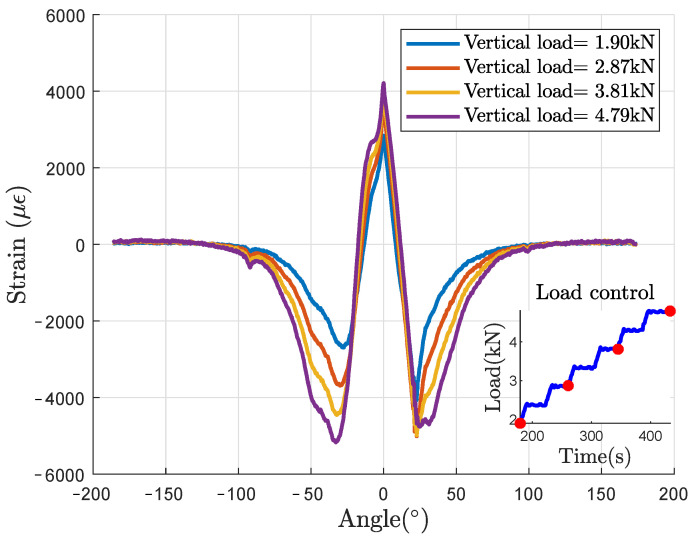
Strain circumferential static profiles, for four vertical loads ($L_{50}$ case).

The typical strain profile obtained from the DOF sensor is represented in Figure 9 for four vertical loads for the $L_{50}$ loading case. The strain profile should be symmetrical relatively to a central vertical axis but some perturbations are observed, mainly due to local rigidity such as tire wear indicators and tread pattern or tire unevenness.

**Figure 10:**
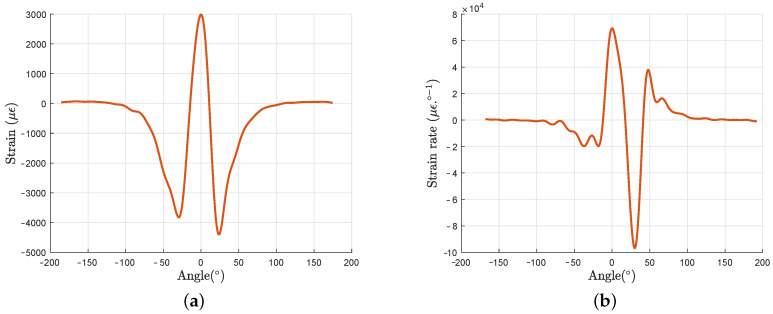
Filtered strain profile (**a**) and its angular derivative (**b**) for a 3000 N vertical load.

The measured strain profile has been filtered with a numerical moving Savitsky-Golay filter to reduce the noise induced on the derivative profile. The filter is defined as smoothing filter of polynomial order 2 and a frame length of 30. The filtered profile and its angular derivative are shown respectively in Figure 10a,b for a 3000 N vertical load.

**Figure 11:**
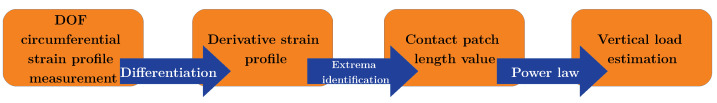
Estimation steps of the load from the circumferential strain measured by DOF.

As can be seen in Figure 11, once the circumferential strain profile is measured, estimation of the load is based on three main steps.

### 4.4. CPL Computation from DOF Strain Data

The first and second steps aim to estimate the length correlated to the contact patch length from the circumferential strain profile. In the following section, the term “contact patch length” will be used to refer to this length.

**Figure 12:**
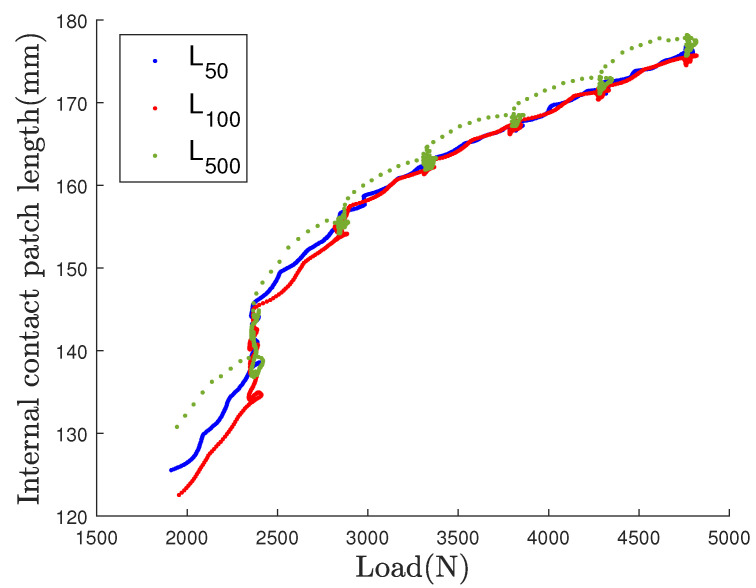
Contact patch length for the three test cases estimated by the peak to peak method from the strain profile derivative.

According to the literature, the peak to peak strain rate method is the most usual way to obtain the contact patch length value. In [28], this method, initiated by Morinaga [29], allows to remove the friction based strain which is combined with the circumferential strain during rolling. Because of a lack of knowledge on friction strain distribution in the case of non-rolling loaded wheel, the peak to peak method is used. The estimated CPL from the strain profile derivative, using the peak to peak distance method, is plotted as a function of the vertical load in Figure 12 for the three loading cases, in the 2000–4800 N load range.

For each cases the observations are the following:

- During the step transition from 2000 N to 2400 N the CPL is highly increasing when reaching and maintaining the 2400 N value, for the three loading cases. This increase of around 10% may be linked to the relaxation of the tire tread rubber, with an applied exceeding the frictional grip force, and resulting to a tread sliding.
- In the following transition step another drifting with a lower magnitude is observed at the beginning of the 2800 N step. It also may be caused by a tread sliding.
- The four loadings from 2800 N to 4800 N are presenting quasi linear relations of the length to the load, for the three loading speeds. CPL is no longer increasing for the periods of steady load; tread sliding seems to not occur again.
- For the higher loading speed $L_{500}$, the CPL values on step transitions adopted a curvilinear motion; increasing and decreasing motion. It is linked to the viscoelastic properties of the rubber/steel structure of the tire.

So we have to distinguish three domains of behavior, below 2400 N, between 2400 N and 2800 N and over 2800 N. Because of these three domains, in order to simplify the relationship between the static CPL and the applied load, the studied load range is fixed between 2800 N and 4800 N. This range usually corresponds to the nominal range of loading on a vehicle wheel.

### 4.5. Load Computation from CPL Data

The third step aims at estimating the vertical load $F_{z}$ from the inner contact patch length $L_{c}$. Based on the previous section observations and from the contact mechanics theory [38], the estimated CPL has a non linear relationship with the vertical load. In this study, it is thus estimated empirically by a power law of the form:

(7) $$F_{z}=\alpha L_{c}^{\beta }$$

**Figure 13:**
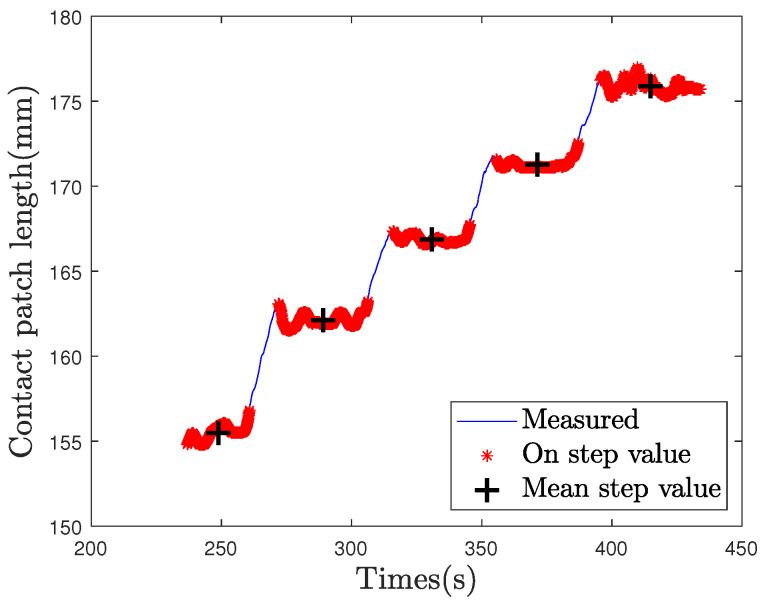
Measured contact patch length (blue); the selected values on steps (red) and the centered mean values of CPL on each step (black cross) used as fitting points.

As explained previously, the studied data range is set between 2800 N and 4800 N. Therefore, the fitting is based on the following equation, including initial values:

(8) $$F_{z}-F_{zi}=\alpha {\left(L_{c}-L_{ci}\right)}^{\beta }$$

with $F_{zi}$ and $L_{ci}$ the initial value of the load and the initial value of contact patch length. The values of parameter α and exponent β are constant and depend on the geometry of the tire and its mechanical behavior. In order to estimate parameters α and β, a logarithmic regression is computed. In the aim of computing the static load, only data recorded during the steps are selected, as highlighted in Figure 13. Finally, the logarithmic regression is done on the mean values of CPL during steps, for the $L_{50}$ loading test, and provides the values of parameters α and β.

The calculated constant values of α and β are $\alpha =1.20\times 10^{-6}$ and β=4.27.

**Figure 14:**
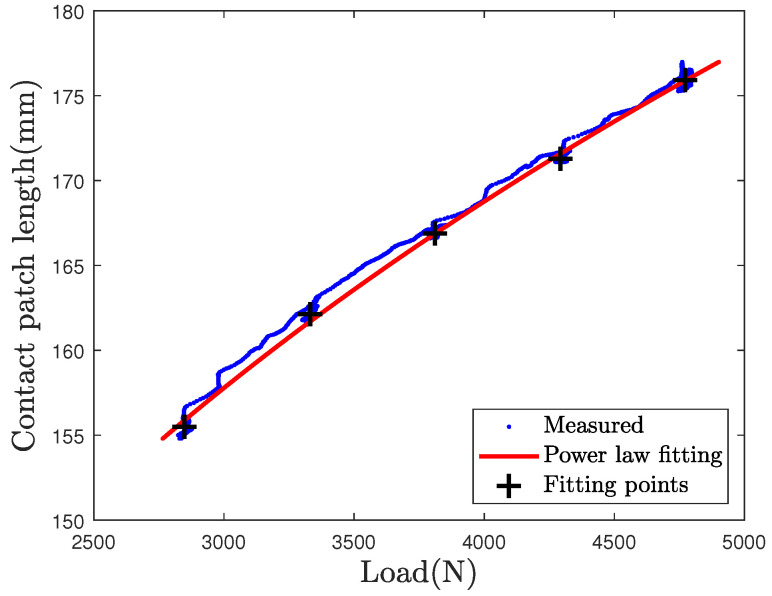
Power law fitting on the $L_{50}$ loading case with $\alpha =1.1998\times 10^{-6}$ and β=4.2756.

The contact patch length and the power law fitting curve for the $L_{50}$ are shown in the Figure 14. Mean values on steps are represented by black crosses on the estimated CPL in blue. The power law fitting curve is plotted in red. It is close to the mean step values. Gaps between the fitting curve and CPL data are observable during step transitions due to dynamic forces.

## 5. Load Estimation from DOFs and Comparison to Laser Estimation

This section presents the load estimation results from the contact patch length. The load estimation based on the contact patch length is involving the power law method as detailed in the previous section. The load estimation based on the deflection from the laser measurement is given as a comparison basis for the newly proposed estimation by an optical fiber. Results are presented through three different angles. Firstly, a time-load representation is made on the $L_{100}$ loading case. Then, the load is estimated from the DOF sensor on the three loading cases and compared to the deflection based load estimation. Finally, a focus is made on the on-step estimated values in order to estimate the accuracy of the experimental in-tire DOF sensor.

### 5.1. Load Estimation for the $L_{100}$ Loading Case

**Figure 15:**
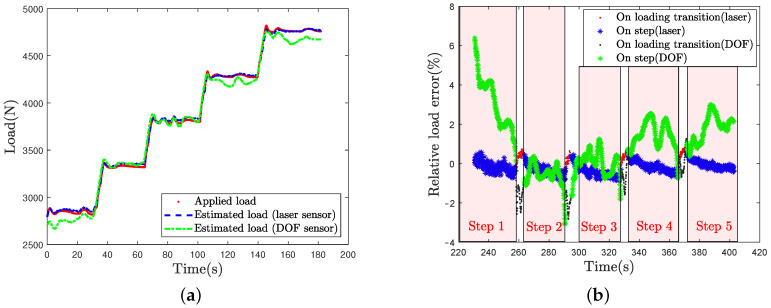
(**a**) Load measurement and estimations from laser and DOF sensors for the $L_{100}$ test case; (**b**) Corresponding relative errors on the load.

Figure 15a represents the applied load and the DOF sensor load estimation as a function of time for the $L_{100}$ loading case. Estimated load by laser is represented as well.

Relative errors for these two estimation methods are given in percents in Figure 15b. The relative error is classically computed as the measured value/estimated value difference over the measured value.

As can be seen on Figure 15a,b, the load estimation by optical fiber presents a higher error (up to 6%) on the first loading step (2800 N). The DOF estimation is exhibiting high error levels around 2% for the two higher loading cases. In the middle range, fluctuations of the load caused by PID servo control on step induce error variations. This error is around 1% for the second and third step. Each loading transition induces an overestimation of the load value around 2 and 3%. Comparatively the laser estimation error is around 1% for each step. A relaxation is observable on each step and can be assigned to the loading setpoint overrun at the beginning of the step and the visco-elasticity effect caused by the sidewalls.

### 5.2. Load Estimation from Dof Sensor and Comparison to the Laser Sensor Estimation

**Figure 16:**
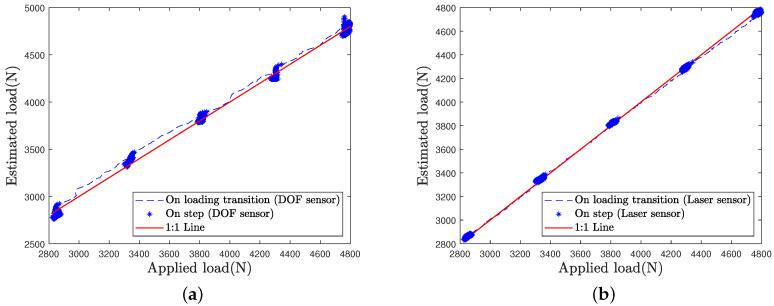
Load estimation for the $L_{50}$ test case: (**a**) DOF sensor (**b**) Laser sensor.

**Figure 17:**
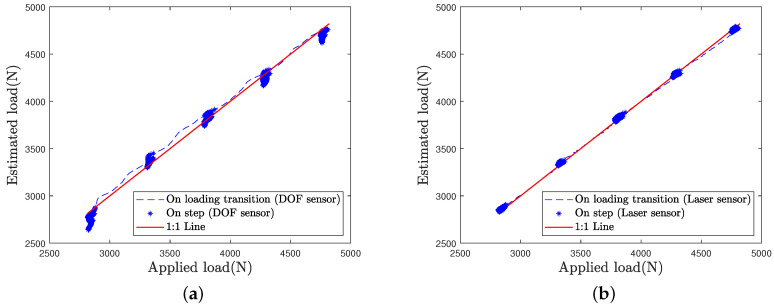
Load estimation for the $L_{100}$ test case: (**a**) DOF sensor (**b**) Laser sensor.

**Figure 18:**
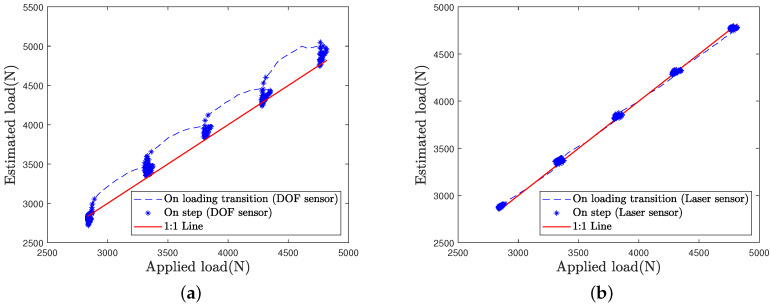
Load estimation for the $L_{500}$ test case: (**a**) DOF sensor (**b**) Laser sensor.

In order to better compare the load estimation for each sensor and each loading case, the estimated load is plotted as a function of the applied load in Figure 16, Figure 17 and Figure 18.

Estimation by the laser sensor appears to be quite insensitive to the loading case, i.e., to the speed of load variations (Figure 16b, Figure 17b and Figure 18b).

Estimation by the DOFS appears to be altered with increasing loading speed (Figure 17a and Figure 18a). Higher errors are visible at load transitions between load steps on Figure 17a and even more on Figure 18a. Nonetheless, DOFS estimation values remain very close to the first bisector line during the load steps.

Higher errors by optical fiber sensor observed during load transitions are due to the tire damping, whose contact length is stretching and then relaxing with the application of a rapid load.

As a confirmation of these graphical observations, the mean absolute percentage error values and the root mean square percentage error (RMSPE) values, computed on steps, are given in Table 4, Table 5 and Table 6. The RMSPE is defined as the square root of the average of squared differences between computed load and measured value. RMSPE induces higher errors than mean absolute error when large errors are encountered.

It is observed that:

- Laser mean relative error has always a value lower than 0.76% and is stable through loading increasing. Its RMSPE follows the same behavior with closed values lower than 0.81% which confirms stability of the load estimation from a deflection measurement.
- DOFS mean absolute percentage error on steps reaches three high values of 3.09% ($L_{100}$ first step), 2.05 ($L_{500}$ first step) and 2.76% ($L_{500}$ second step), but other values are lower than 2%.
- RMSPE and mean absolute percentage error value shows that both methods are close but with a higher dispersion for DOF sensor based method.

## 6. Conclusions

This work is motivated by the fact that wheel load estimation is of importance both for infrastructure resilience and for vehicle safety concerns. The bibliography indicates that for a still vehicle existing methods as laser impose a wheel rotation until the sensors points towards the tire contact patch. Moreover on-rolling load estimation remains hard due to impact forces.

The novelty of this research is to investigate a distributed sensor, able to estimate the static wheel load, whatever the wheel angle position.

Experiments have been carried out with a static tire loaded by a hydraulic press. The proposed new estimation mean of the load, a Distributed Optical Fiber Sensor, is used for these experiments in addition to an embedded laser sensor as a comparison basis. These two estimation means are respectively involving contact patch length and tire deflection evaluations.

For several applied loads from 2800 N to 4800 N that corresponds to the tire load working range, DOF sensor load estimation is realized with a mean absolute percentage error on the range of 1.13%, but with punctual errors up to 3.09% in conditions of low or high loads in the experimented range.

Loading speed increasing induces more errors of the load estimation. Indeed three loading speeds have been investigated in order to estimate the impact of the tire characteristics like viscoelasticity on the measurements. However this work is focusing on a static tire, as the load knowledge for a still vehicle, before starting a journey, is already a useful information. So a perspective for a moving vehicle exists but should require dynamic corrections as well as technical adaptations like resistant fiber bonding to tire dynamics and sensor interrogator embedded in the wheel or linked to the sensor by a rotary coupler.

The laser-based estimation of the vertical load is realized with a mean absolute error of 0.29%, with much less dispersion, the maximum error being of only 0.76%.

Load processing from DOF sensor is less accurate than the laser sensor. The higher number of in the DOF sensor based load estimation, may induce more errors. Moreover, the DOF sensor is more sensitive to the speed of loading transitions than the laser. Nevertheless load estimation from the DOF sensor remains close to the applied load in each case.

From a technical point of view, optical fiber sensor offers great performances and is spaced-saving. Nevertheless, it remains fragile even if more robust optical fiber, used for structure health monitoring can be more robust than the used one. In-tire integration involves an efficient bonding. The current issue of optical fiber in tire is the acquisition system which has to be miniaturized to be embedded in a vehicle.

As a conclusion, the new estimation mean, involving a Distributed Optical Fiber Sensor, has the advantage of the load estimation regardless of the wheel angular position. In perspective, the developed methodology based on that mean could be used for continuous wheel load estimation for moving vehicles, benefiting to control and safety vehicle systems.

## Figures and Tables

**Table 1:** Wheel-embedded sensor type and aim of some tire deflection studies.

Reference	Sensor Type	Aim
[12]	Ultrasonic sensor	Proof of concept, radial displacement profile sensing and deflection-load relationship identification
[13]	Three aligned ultrasonic sensors	Deflection sensing, identification of CPL and speed, load and pressure influence, radial stiffness estimation
[14]	Laser	Proof of concept, radial displacement profile sensing, influence quantities identification
[15]	Laser	CPL identification and behavior through different pressure, speed and load
[16]	IR - PSD	Deflection sensing, deflection-load relationship

**Table 2:** Major literature references on the strain/wheel load relationship.

Reference	Strain Direction Circumferential (C), Transversal (T)	Estimated Characteristic	Method/Model	Sensor or Evaluation Mean	Vertical Load Relationship
[19,24]	C	CPL	Differentiation and peak to peak	FBG	Not defined
[20]	C	CPL	Differentiation and peak to peak	Gauges	Not defined
[25]	C	Radial displacement and CPL	Flexible ring model (displacement) and peak to peak	Strain gauges	Linear relationship
[26]	C/ T	Strain value and CPL	Differentiation and peak to peak (CPL identification)	FBG	Not defined
[27]	C	Displacement	Flexible ring model on viscous foundation	Gauges	Flexible ring model
[28]	C	CPL and strain value	Differentiation peak to peak	FEM (simulation)	Integral of circumferential strain

**Table 3:** Major literature references on acceleration measurements/wheel load relationship.

Reference	Acceleration Direction: Radial (R), Circumferential (C)	Estimated Characteristic	Method	Sensor	Vertical Load Relationship
[30]	C	CPL	Peak to peak	Tri-axial	Flexible ring model
[31]	R	CPL and Radial max displacement (Comparison)	Zero crossing(CPL)/Double integration	Tri-axial	Recursive least square fitting
[32]	R	CPL	Zero crossing acceleration profile	One axis accelerometer	Identification of correlation between CPL and load
[33]	R/C	CPL	R.: Zero crossing/C.: Peak to peak	Tri-axial accelerometer	Identification of linear relationship (load vs CPL)
[34]	C	CPL	Peak to peak	Tri-axial	Calibration

**Table 4:** Loading case with low speed force variation between steps ($L_{50}$).

Step Load	Applied (N)	Mean Absolute Percentage Error (Laser Sensor)	Mean Absolute Percentage Error (DOFs)	RMSPE (Laser Sensor)	RMSPE (DOFs)
1	2850	0.28	1.14	0.35	1.41
2	3330	0.21	1.16	0.26	1.30
3	3810	0.25	0.40	0.30	0.50
4	4290	0.15	0.83	0.19	0.91
5	4770	0.19	0.80	0.26	0.95

**Table 5:** Loading case with medium speed force variation between steps ($L_{100})$.

Step Load	Applied (N)	Mean Absolute Percentage Error (Laser Sensor)	Mean Absolute Percentage Error (DOFs)	RMSPE (Laser Sensor)	RMSPE (DOFs)
1	2840	0.27	3.09	0.32	3.34
2	3320	0.36	0.63	0.41	0.80
3	3810	0.46	0.48	0.51	0.60
4	4280	0.25	1.48	0.30	1.60
5	4770	0.18	1.77	0.21	1.89

**Table 6:** Loading case with high speed force variation between steps ($L_{500})$.

Step Load	Applied (N)	Mean Absolute Percentage Error (Laser Sensor)	Mean Absolute Percentage Error (DOFs)	RMS Percentage Error (Laser Sensor)	RMS Percentage Error (DOFs)
1	2850	0.43	2.05	0.50	2.45
2	3330	0.66	2.76	0.72	3.20
3	3810	0.76	1.64	0.81	1.87
4	4290	0.46	0.67	0.51	1.16
5	4770	0.30	1.51	0.35	1.80

## Data Availability

Not available.
